# Altered Circadian Rhythm and Metabolic Gene Profile in Rats Subjected to Advanced Light Phase Shifts

**DOI:** 10.1371/journal.pone.0122570

**Published:** 2015-04-02

**Authors:** Laura Herrero, Lorea Valcarcel, Crhistiane Andressa da Silva, Nerea Albert, Antoni Diez-Noguera, Trinitat Cambras, Dolors Serra

**Affiliations:** 1 Department of Biochemistry and Molecular Biology, Facultat de Farmàcia, Institut de Biomedicina de la Universitat de Barcelona (IBUB), Universitat de Barcelona, Barcelona, Spain; 2 Centro de Investigación Biomédica en Red de Fisiopatología de la Obesidad y la Nutrición (CIBEROBN), Instituto de Salud Carlos III, Madrid, Spain; 3 Department of Physiology, Facultat de Farmàcia, Universitat de Barcelona, Barcelona, Spain; 4 Laboratório de Neurobiologia e Ritimicidade Biológica, Departamento de Fisiologia, Universidade Federal do Rio Grande do Norte, Natal, Brasil; University of Alabama at Birmingham, UNITED STATES

## Abstract

The circadian clock regulates metabolic homeostasis and its disruption predisposes to obesity and other metabolic diseases. However, the effect of phase shifts on metabolism is not completely understood. We examined whether alterations in the circadian rhythm caused by phase shifts induce metabolic changes in crucial genes that would predispose to obesity. Three-month-old rats were maintained on a standard diet under lighting conditions with chronic phase shifts consisting of advances, delays or advances plus delays. Serum leptin, insulin and glucose levels decreased only in rats subjected to advances. The expression of the clock gene Bmal 1 increased in the hypothalamus, white adipose tissue (WAT), brown adipose tissue (BAT) and liver of the advanced group compared to control rats. The advanced group showed an increase in hypothalamic AgRP and NPY mRNA, and their lipid metabolism gene profile was altered in liver, WAT and BAT. WAT showed an increase in inflammation and ER stress and brown adipocytes suffered a brown-to-white transformation and decreased UCP-1 expression. Our results indicate that chronic phase advances lead to significant changes in neuropeptides, lipid metabolism, inflammation and ER stress gene profile in metabolically relevant tissues such as the hypothalamus, liver, WAT and BAT. This highlights a link between alteration of the circadian rhythm and metabolism at the transcriptional level.

## Introduction

Obesity is acquiring epidemic proportions, increasing its prevalence in developing countries and occurring at younger ages. This presents a major public health concern and is associated with other chronic diseases such as type 2 diabetes, cardiovascular disease, hypertension, hypercholesterolemia, hypertriglyceridemia, arthritis, asthma, and certain forms of cancer [1]. In addition, circadian disruption is associated with the onset of metabolic syndrome, obesity and type2 diabetes [2].

Circadian clocks are time-tracking systems that enable organisms to anticipate environmental changes, thereby adapting their behavior and physiology to the time of day [3]. In mammals, the master clock in the suprachiasmatic nucleus (SCN) of the hypothalamus generates circadian rhythms, also synchronizing peripheral oscillators in almost all cell types and tissues. Thus, dysfunction of the circadian clock can have significant downstream effects in different cell types and organ systems [4]. Entrainment to environmental cycles such as the external light-dark cycle takes place due to photic information travelling directly from light sensitive ganglion cells in the retina to the SCN [5]. In this way, the internal clock is entrained to the external cycles and the rhythm of the organism acquires the same period as the rhythmic environment. This ensures that physiological and behavioural processes will ensue at the best time for the individual.

At the molecular level, the circadian clock is formed by a group of genes that are transcribed periodically, and which are interconnected in transcriptional, translational, and post-translational modification loops. This auto-regulates the clock system and also regulates the circadian expression of output genes. The genes encoding the core clock mechanisms include *Clock*, *Bmal1*, *Period 1–3* (*Per1*, *Per2* and *Per3*), and *Cryptochrome 1–2* (*Cry1* and *Cry2*). The autoregulatory transcription-translation loop formed by CLOCK:BMAL1 and PER-CRY constitutes the core clock and generates 24h rhythms of gene expression [6].

Phase shifts are quite common in modern society due to jet-lag, shift work, sleep disruption and alterations in food consumption. The experimental induction of jet-lag in rodents produces a deleterious effect on lifespan, and advances in the light/dark cycle have a stronger effect on organisms than delays [7,8]. Moreover, complete elimination of the circadian rhythm has been related to metabolic syndromes, obesity and diabetes [2,9]. The circadian clock regulates metabolism in peripheral tissues by mediating the expression or activity of certain key metabolic factors, and many molecular candidates have been proposed as links between the circadian clock and metabolic tissues such as SIRT1 and the PPAR family [10,11]. Moreover, nutrient sensors relay information on cell nutrient status to the circadian clock [12]. Despite the large body of evidence associating circadian disruption and obesity-induced metabolic defects the underlying mechanism remains unclear.

Here we hypothesize that alterations in the circadian rhythm of rats by phase shifts induce metabolic changes in crucial genes that would predispose to obesity. We found that rats under standard chow subjected to lighting schedules with chronic phase advances had lower fasting blood glucose, insulin and leptin levels. In addition, the expression of circadian rhythm and lipid metabolism genes were altered in liver, white and brown adipose tissue (WAT and BAT, respectively). This highlights the important link between alteration of the circadian rhythm and metabolism at the transcriptional level.

## Materials and Methods

### Animals

Study animals were male Wistar rats (Charles River, France) with free access to water and chow. They were weighed every two weeks throughout the experiments to monitor their health. All procedures complied with the institutional guidelines for the care and use of laboratory animals established by the Ethical Committee for Animal Experimentation at the University of Barcelona.

### Phase shifts

Since weaning, rats had always been under 24h light-dark cycles (12h light:12h darkness) and maintained in individual cages sized 25x25x15 cm. When rats were 70 days old (day 0 of the experiment) and thus, they had a stable circadian rhythm [13], they were distributed at random into 4 groups of 8 animals each, which were maintained in separate sound-proof and temperature controlled rooms, under different lighting conditions:

- 24h light-dark cycles (12h light:12h darkness) (control)
- 24h light-dark cycles with phase advances of 6h every 5 days (Advanced)
- 24h light-dark cycles with phase delays of 6h every 5 days (Delayed)
- 24h light-dark cycles with phase shifts every 5 days, alternating phase advances and delays (Advanced-Delayed).

Phases were advanced by shortening the light period and the dark period by 3h every five days (this implies four 12:12 light-dark cycles and one cycle of 9:9h). Phases were delayed by lengthening the light period and the dark period by 3h every five days (this implies four 12:12 light-dark cycles and one cycle of 15:15h). Rats were maintained under these conditions for 67 days, until the circadian patterns induced by light were noticeable and stable. Light intensity at the level of cages was 500 lux during the light phase and less than 0.5 lux of dim red light during the dark stage. Light was provided by fluorescent lamps facing the wall in front of the animals’ cage in order to avoid direct exposure to the lamp. On day 67 of the experiment, rats were fasted for 8–10h and then killed by decapitation under isoflurane anaesthesia at the beginning of the dark phase, which coincided with the onset of the main activity phase with all the animals. Samples of liver, hypothalamus, epididymal WAT and interscapular BAT were obtained.

### Motor activity

The motor activity of each animal was recorded by means of activity meters that used two perpendicular crossed infrared beams situated 6 cm above the floor of the cage. Each beam interruption represented an activity count that was registered and compiled in 15 min intervals, obtaining time series of motor activity. Light/Dark (LD) cycles were recorded simultaneously to the activity in a separate channel connected to a photocell pulse generator. To study the circadian pattern, motor activity data were represented in double-plotted actograms at modulo 24h, and a Chi squared periodogram [26] was calculated to determine the main circadian periodicity. The scanning range for the periodogram was from 20h to 26h in steps of 5 min, and the level of significance was p = 0.05, with Bonferroni’s correction for multiple estimations. Periodogram analysis provided the value of the significant periods in the data series and the percentage of variance explained by these rhythms (PV). PV can be used as an indirect measure of the daily phase stability and as an indicator of the significance of the rhythm, since greater PV indicates repetitive and stable patterns on a daily basis.

### Measurement of serum metabolites

On day 40 of the experiment, blood samples (between 40 and 50 μl) were obtained by gentle section of the tail tip and collected in a capillary tube (Microvette CD300 K2E) at the beginning of the dark phase (between ZT12 and ZT14) for each group of rats and after 10–12h of fasting. This circadian time was chosen since in a previous experiment (S1 Fig.) with control rats and those submitted to advances, we found differences in leptin concentrations between the two groups only at this time point. Serum was extracted and frozen in aliquots. Blood glucose concentrations were measured using a Glucometer ELITETM (Bayer). ELISA was used to measure insulin (Millipore MEZRMI-13K), leptin (Millipore EZRL-83K) and FGF21 (Biovendor, Cat.No.RD291108200R). Serum triacylglyceride levels were measured with a kit from Sigma (TR0100-1KT), non-esterified fatty acid levels with a kit from WAKO (Ref: 434–91795 and 436–91995), and cholesterol was measured with a kit from Linear Chemicals S.L (Ref: 1118005).

### RNA Extraction and Quantitative RT-PCR

Total RNA was extracted from rats between ZT12 and ZT14 from frozen, pulverized tissue using RNeasy (QIAGEN). cDNA was synthesized with the Transcriptor First Strand cDNA Synthesis kit (Roche). PCR amplifications were performed using intron-skipping primers and SYBR Green I Master kit (Roche) and measured with the Roche LightCycler 480 II. All primers sequences are available upon request. Real-Time PCR System was normalized against S18 and TBP.

### Histology

Histological examination was done using 4 μm thick formalin-fixed, paraffin-embedded tissue sections stained with haematoxylin and eosin (H&E) at the Pathology Department of the Hospital Clinic of Barcelona.

### Statistical analysis

Motor activity time series were analysed using the integrated package for chronobiology ‘‘El Temps” v.251 (A. Díez-Noguera, Universitat de Barcelona, 2011). Statistical analysis was performed with the SPSS 18.0 package. All figures and statistical analyses were generated using GraphPad Prism 6 and Adobe Illustrator. Data are expressed as the mean ± SEM and analysed statistically using Student’s *t*-test or ANOVA. *p*< 0.05 was considered statistically significant. Data available upon request.

## Results and Discussion

### Rhythmic profiles, body weight, motor activity and serum metabolites in rats under different light shifts

**Fig 1:**
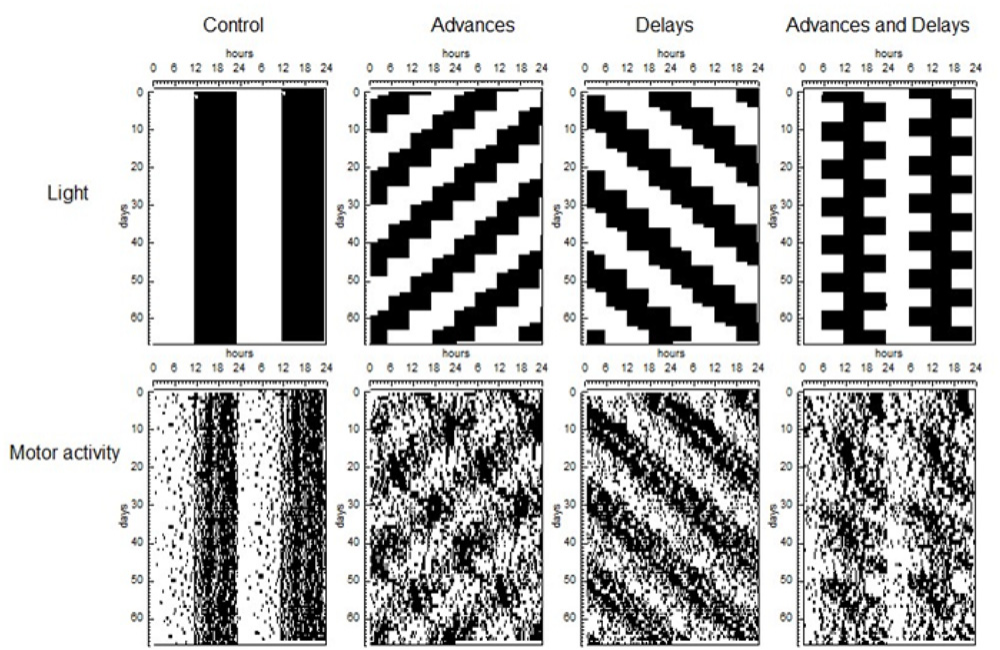
Lighting and motor activity patterns. Top: Double plotted graphs corresponding to light patterns, where the dark areas correspond to darkness. Bottom: Motor activity of a representative animal of each group.

To compare the global rhythmic pattern of each animal, we analysed periodograms for the same data set. Results show that motor activity patterns of the rats differed according to lighting conditions (Fig. 1). As expected [13], the motor activity rhythm of the advanced group was the most disturbed by the phase shifts. Both control and delayed groups showed a well-defined circadian rhythm, with periods of 24h and 24h 70 min, respectively, which explain 25% (SEM 1.3) of the variance of the data series. In the advanced group, chronic phase shifting was indicated by the presence of two simultaneous rhythms with different period: one whose period equals that of the light pattern (22h 50 minutes) and explained 7.7% (SEM 1.2) of the variance, and a free running rhythm with a period of 25h 8 min (SEM 4.3 min) that explained 5.48% (SEM 0.6%) of the variance. Similarly in the case of advanced and delayed group, all the animals showed a rhythm of 24h, which explained 14.6% of the variance (SEM 1.3) and in addition, 7 out of 8 rats showed a significant rhythm with a mean value of 25h 4min (SEM 2.23), not driven by light, which explained 4.63% (SEM 0.4) of the variance and can be considered the consequence of the masking effects of the phase shifts. The percentage of variance explained by the light-dependent rhythm differed among the groups, suggesting different importance of this rhythm on the animal behaviour: ANOVA and posthoc tests indicate that the PV of the advance group was lower than that of advances and delays group (p<0.05), delays group (p<0.001) and controls (p<0.001) and that the PV of advances and delays group was also lower than delay and control groups (p<0.05 in both cases).

**Fig 2:**
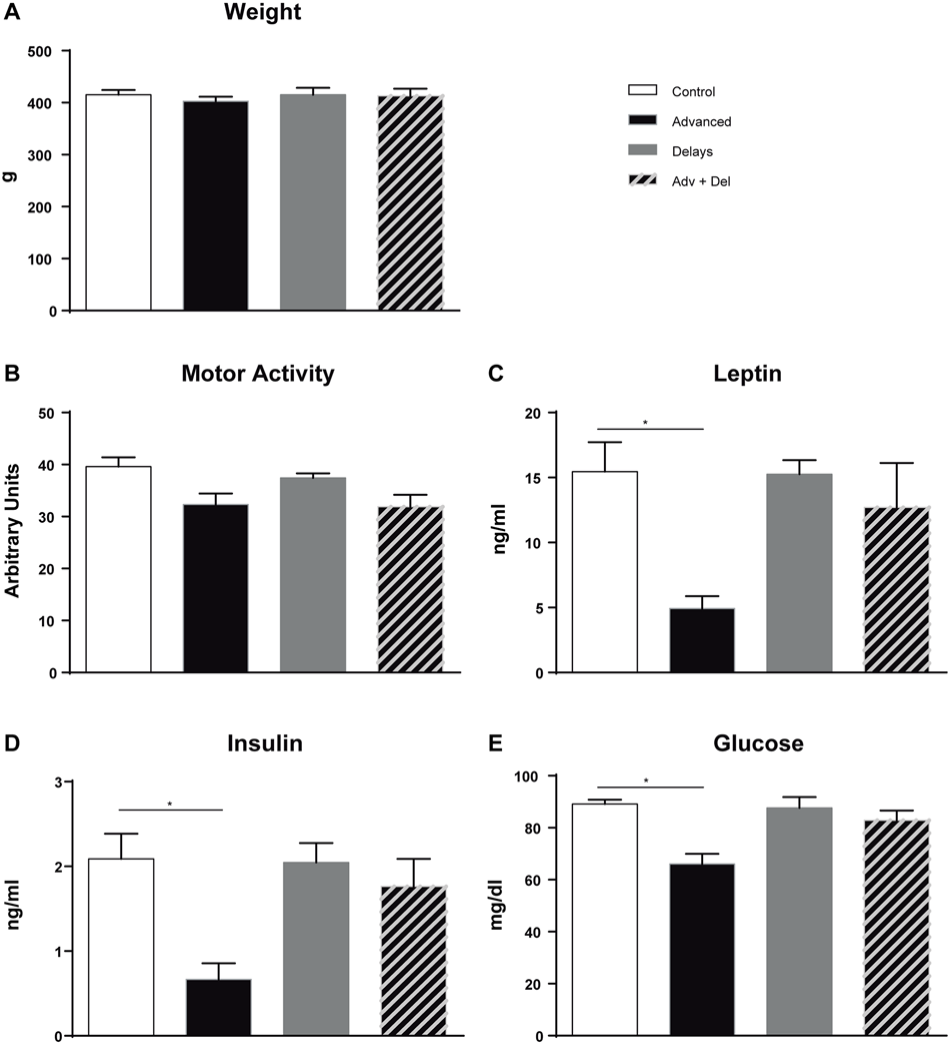
Weight, motor activity and fasting blood glucose, insulin and leptin levels. Adv + Del: Advanced and Delayed group. (*p<0.05, n = 8).

Although no differences were seen in body weight or motor activity between the groups, fasting blood glucose, insulin and leptin levels decreased only in the advanced group and at the onset of darkness (Fig. 2). This coincides with results from a previous experiment, in which serum leptin was also measured in chronic advanced and control rats at different collection times (S1 Fig.) and leptin levels were only reduced at the onset of darkness. We thus continued our metabolic study with the control and advanced group. Among these two groups, no differences were seen in serum FGF21, NEFA, cholesterol, or triacylglyceride levels (S1 Table).

### Circadian clock gene expression is altered in rats with phase advances in the hypothalamus, BAT, WAT and liver

**Fig 3:**
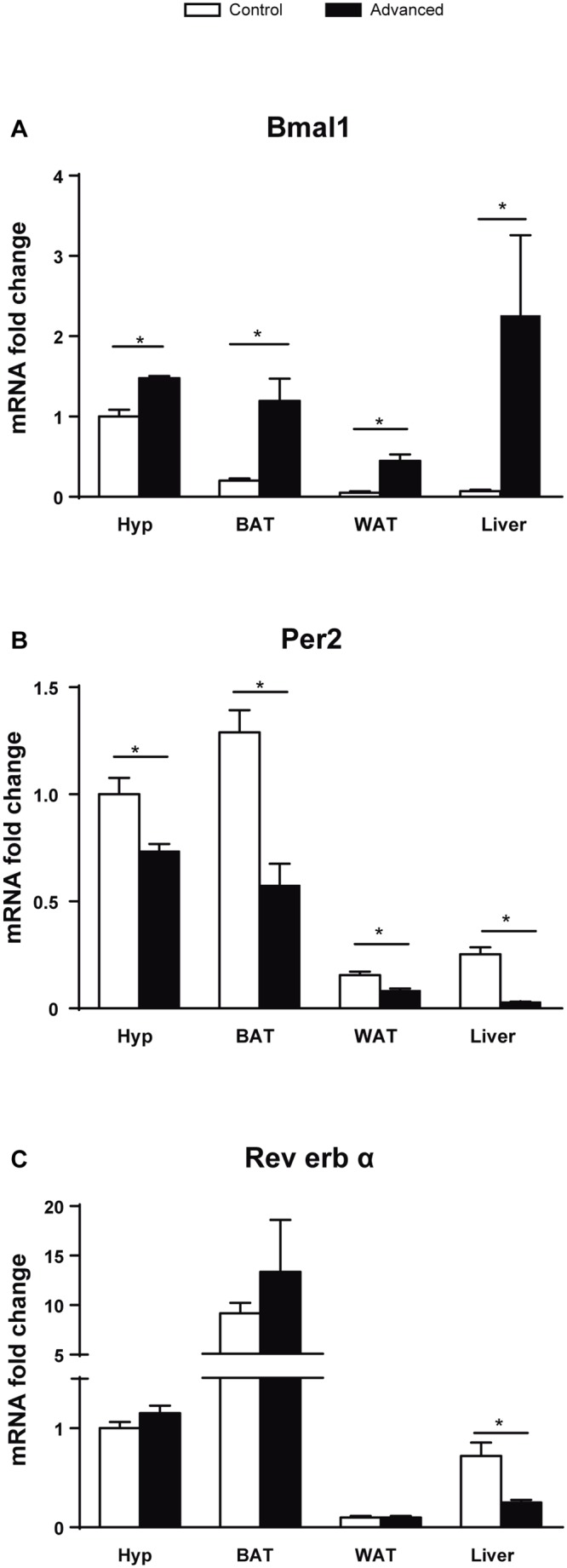
Clock genes in hypothalamus, liver, WAT and BAT. mRNA expression of genes involved in the core clock system. (*p<0.05, n = 7–8).

The transcript levels of the clock genes *Bmal1*, *Per2* and *Rev-erbα* were examined in the hypothalamus, WAT, BAT and liver (Fig. 3). In all these tissues the expression of *Bmal1* was increased and *Per2* was decreased compared to control animals (Fig. 3A and B). The mRNA expression of *Rev-erbα* did not change in hypothalamus, BAT or WAT, whereas a significant decrease was seen in liver (Fig. 3C). We also observed that, in control animals, the mRNA levels of *Bmal1* were highest in the hypothalamus, while *Per2* and *Rev-erbα* mRNA levels were highest in BAT.

### Advanced light shifts altered gene expression in liver and hypothalamus

**Fig 4:**
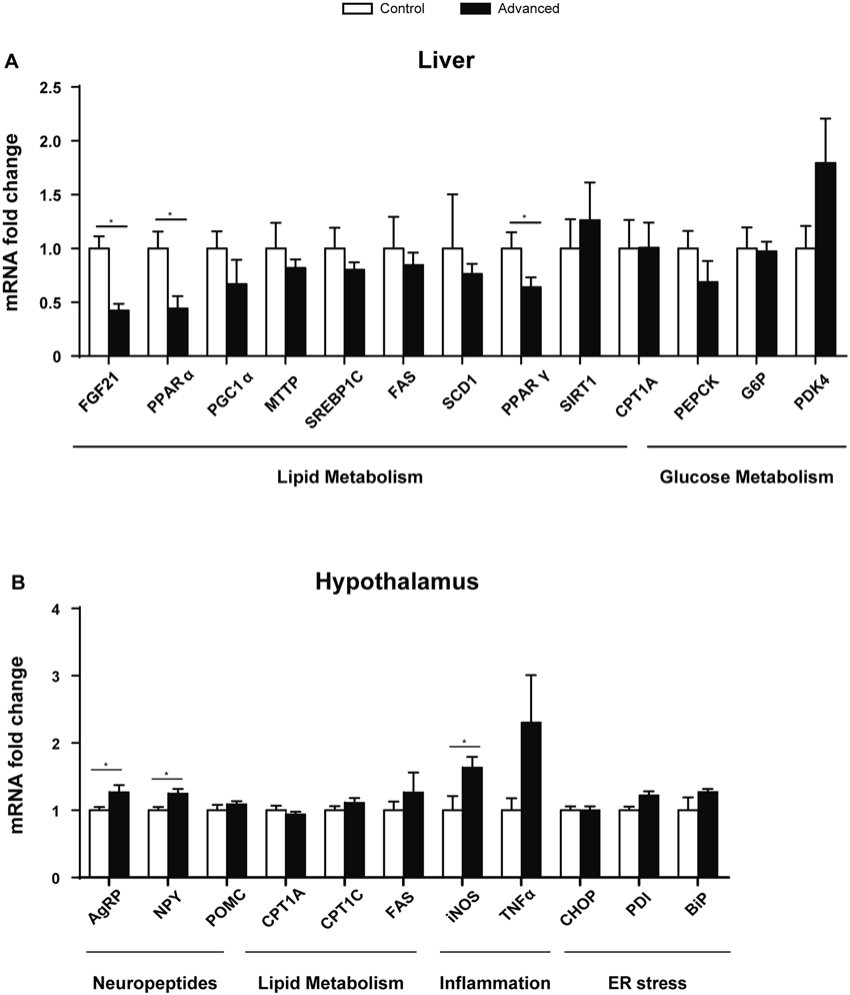
Gene expression analysis in liver and hypothalamus. (A) Expression of genes involved in lipid and glucose metabolism in the liver. (B) Lipid metabolism, inflammation, neuropeptides and Endoplasmic Reticulum (ER) stress genes were analysed in the hypothalamus. (*p<0.05, n = 7–8).

To examine the alterations in metabolism, we analysed genes involved in lipid and glucose metabolism in the liver. FGF21 regulates hepatic lipid metabolism during fasting and its expression is positively regulated by PPARα. PPARγ activation has positive effects in glycemic control, insulin resistance, lipid metabolism and inflammation. A decrease in the expression of *Fgf21* (*p* = 0.017), *Pparα* (*p* = 0.0371) and *Pparγ* (*p* = 0.05) was observed in the liver (Fig. 4A). No changes were observed in other genes involved in lipid and glucose metabolism.

The melanocortin system, comprising anorexigenic pro-opiomelanocortin (POMC) expressing neurons and orexigenic agouti-related protein (AgRP)/ neuropeptide Y (NPY) co-expressing neurons, is crucial for normal energy homeostasis. AgRP is co-expressed with NPY, increasing appetite and decreasing metabolism and energy expenditure. A higher expression of both neuropeptides, *Agrp* and *Npy*, was observed in the hypothalamus (Fig. 4B). Moreover, an increase in the inducible nitric oxide synthase (*Inos*) gene was observed, indicating potential inflammation in the tissue (Fig. 4B). No differences were seen in neuronal nitric oxide synthase (*nNOS*; S3 Fig.).

### Advanced light shifts altered BAT and WAT gene expression

**Fig 5:**
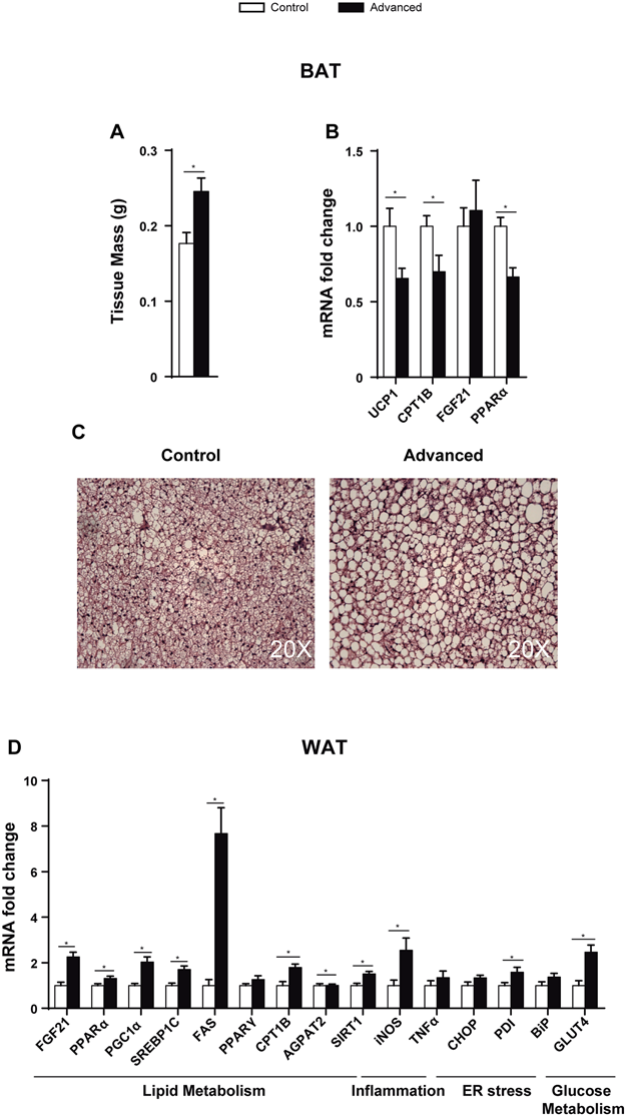
Advanced light shifts lead to histological and gene expression changes in BAT and WAT. BAT (A) mass, (B) mRNA expression and (C) histology (H&E staining) of advanced and control rats. (D) WAT mRNA gene expression. (* = p<0.05, n = 7–8).

Although no differences were seen in WAT weight (advances: 3.3 +/- 0.29 g; control: 3.7 +/- 0.30 g), BAT mass was increased in the advanced group and histological examination showed an accumulation of lipids, indicating a brown-to-white transformation similar to that found in obesity (Fig. 5A and C). No histological changes were seen in any other tissue analysed (S2 Fig.). Accordingly, the uncoupling protein 1 (*Ucp-1*) gene, which is a BAT marker of thermogenesis, decreased its expression by 40% (*p* = 0.041) (Fig. 5B). Furthermore, *Cpt1B* (*p* = 0.038) and *Pparα* (*p* = 0.003) were also downregulated in the advanced group (Fig. 5B), indicating that lipid metabolism is not activated in BAT of advanced rats.

The expression of genes involved in both lipid oxidation (*Cpt1B*, *Fgf21*, *Pparα* and *Pgc1α*) and synthesis (*Srebp1C*, *Agpat2*, *Fas* and *Sirt1*) was increased in WAT of the advanced group (Fig. 5D). We also found an increase in the mRNA expression of the inflammatory marker *Inos* (*p* = 0.009) and *Pdi* (*p* = 0.044), a protein disulfide isomerase that folds the proteins that are accumulated due to ER stress. The expression of the insulin-dependent glucose transporter 4 (Glut4) was increased in the advanced group (Fig. 5D).

## Conclusions

Organisms have strong advantages when their biological rhythms match and maintain a phase relationship with the environmental cycles. The circadian systems are linked to the light/dark and nutrient environment by a network of local tissue specific clocks in both the brain and periphery. This combination is achieved by a group of genes that act in feedback loops and integrate the circadian clock oscillations and metabolism [12]. Although there is evidence of the relation between circadian rhythm disruption and obesity, the mechanisms involved are unclear. The difficulties arise from the tissue specific action of the genes involved in circadian rhythm and metabolism, but also from several other factors, such as the nutrient status and meal timing, which by themselves alter the circadian system [14].

In previous studies, animals treated with high-fat diet (HFD) showed a disruption in behavioural and molecular circadian rhythms, higher activity during the light phase, and an increase in food consumption [15]. In our case, we submitted young rats, fed with a standard diet, to 8 weeks of phase shifts (advances, delays and advances + delays). Body weight was maintained in all groups. This was not surprising since rats were fed normal chow and phase shifts were applied during a short period of time. Interestingly, at this time point serum glucose, insulin and leptin levels were already decreased but only in the advanced group. We thus hypothesized that the decrease of leptin could induce orexigenic neuropeptides in the hypothalamus and be the first signal of possible alterations at the transcriptional level in metabolic pathways. We cannot rule out that the other groups of rats submitted to phase shifts (delays and advances + delays) might also have manifested some alterations in metabolism. However, we decided to focus on the analysis of the group that was most likely to show alterations: the advanced group, which shows the most altered rhythm. Leptin acts as a satiety signal and inhibits NPY and AgRP [16]. The mRNA levels of both neuropeptides increased in the hypothalamus in the advanced group. This fits with the low levels of leptin observed in the advanced group and suggests modifications in the regulation of feeding. Other studies based on sleep time restriction also showed altered leptin levels in WAT resulting in deregulation of adipose metabolism [17]. Chronic advances produce dissociation in the circadian system, which implies that rats may also feed in the light period [18][19]. Although the feeding pattern and body temperature was not measured in our study, alterations in food intake may underpin alterations in serum glucose, insulin, and leptin. Thus, the discrepancy between light-dark cycles and food intake might have contributed to the alterations (*i*.*e*. insulin or leptin) found in our study. Further experiments should clarify what would happen under chronic phase advances and food restricted to darkness.

The rhythmic behaviour of the chronic advanced group was different from the rest. This group showed most alterations in the manifestation of the motor activity rhythm, resembling the pattern observed when rats were submitted to LD cycles with a period shorter than 24h with the presence of two simultaneous circadian rhythms with different period [20,21]. Since chronic delays produced only a single rhythm with a PV similar to that of controls, we suggest that the cause of the metabolic alterations are not the phase shifts by themselves but the dissociation of the circadian rhythm. Changes in both Bmal1 and Per2 mRNA levels in all tissues studied, at the onset of darkness (and onset of activity), of the advanced group compared with controls indicate an alteration of the circadian system. This coincides with other models of circadian disruption found with different phases shifts (*e*.*g*. rats submitted to LD mimicking the rotating shift-work with 8h phase delay every second day [22] or reversing the photoperiod every 3–4 days [23]).

All our results are referred to the onset of darkness, which limits our experiment, since many metabolites have circadian expression throughout the day. The lack of variation in cholesterol, triacylglycerides and NEFA serum levels of the advanced group may have been due to the serum collection time (2h after the start of the dark phase) and is consistent with the findings of Pan *et al*. [24]. These authors showed that triacylglicerides and cholesterol levels did not change in *clock* deficient mice at the onset of the dark phase. In addition, we did not observe changes in FGF21 serum levels between control and the advanced group. Although we have seen a decrease in liver FGF21 mRNA levels, which is considered the main FGF21 secretory tissue, other metabolic tissues such as WAT, BAT, skeletal muscle and pancreas might have contributed to the lack of differences in FGF21 serum levels [25,26]. Moreover, other changes seen in the present study such as the differences in BAT weight are unlikely to vary throughout the day. The advanced group showed a decrease in glucose levels, which could mean an initial alteration of glucose metabolism due to chronic phase shifting. While some studies in *Bmal1* knockout mice showed alterations in glucose metabolism [27,28], a recent paper based on *Bmal1* knockout mice did not report glucose alterations in serum [29]. It has been observed that *Clock* and *Bmal1* knockouts in mouse pancreas produced hypoinsulinaemia and diabetes [30]. Although the expression of BMAL1 increased in the advanced group of rats, we hypothesize that either up- or down-regulation of the circadian rhythm genes may affect insulin production and signalling. Thus, despite serum insulin was decreased, *Glut4* was overexpressed in WAT, suggesting a deregulation of the insulin signalling pathway.

PPARα is involved in fatty acid oxidation and regulates FGF21 production in the liver. Fasting or mice treatment with the PPARα agonist Wy-14,643 induces FGF21 mRNA expression [31][32]. On the contrary, PPARα deficient mice showed decreased FGF21 mRNA levels and fasting or PPARα agonist treatment did not induce FGF21 mRNA expression [32]. Although both control and the advanced group of rats were fasted for 8–10h before sacrifice, the advanced group showed a decrease in liver *Fgf21* and *Pparα* suggesting a predisposition to obesity. It is worth noticing that the same factors that decreased in liver, increased in WAT, indicating tissue specificity.

In rats subjected to advanced phase shifts, BAT mass was increased and histological examination showed a brown-to-white transformation with lipid accumulation. The expression of the BAT differentiation marker *Ucp1* decreased as well as that of *Fgf21*, which is expressed during thermogenic activation [25]. Moreover, *Cpt1B* mRNA expression was decreased, indicating reduced lipid oxidation. BAT is present and active in adult humans and decreases in obesity and type 2 diabetes [33]. Brown adipocytes enhance energy expenditure, increasing glucose and fatty acid uptake. Thus, activation of BAT might be a potential tool to counter obesity [34]. According to this, the loss of BAT differentiation in the advanced group of animals reflects an initial state of obesity.

The expression of lipid metabolic genes in WAT was strongly altered in the advanced group. Lipid oxidation (*Cpt1B*, *Fgf21*, *Pparα* and *Pgc1α*) and synthesis (*Srebp1C*, *Fas* and *Agpat2*) were increased. *Pparα* has been implicated in obesity and metabolic diseases [14] while *Pgc1α* plays an important role in the crosstalk between circadian clock circuitry and metabolism [35]. It is noteworthy that in previous studies animals treated with HFD showed a decrease in WAT BMAL1 and SIRT1 [36], while in our case animals were under chow diet and both were up-regulated. This indicates that the consequences of diet in lipid metabolism are different from the effects produced by circadian rhythm alterations.

Metabolic syndrome is associated with chronic low-grade inflammation and increased ER and ROS damage [37]. Inflammation has also been reported in the hypothalamus at the initial state of obesity [38]. Our results show that the expression of markers of inflammation and ER stress were increased in WAT (*Inos* and *Pdi*) and hypothalamus (*Inos*) of advanced rats.

In summary, our experiments showed alterations in metabolic, inflammatory and ER stress factors in young animals submitted to chronic advanced phase shifts without diet manipulations such as restricted feeding or HFD. Our study also questions the ability of young subjects to cope with phase shifts and highlights the role of stable circadian rhythms in the prevention of metabolic diseases. Although further studies in humans are necessary to extrapolate the present data, our results suggest the need to assess the direction of the shift work schedules in order to prevent metabolic alterations and their pathological consequences.

## Supporting Information

S1 Fig Leptin levels in advanced and control rats at different collection times. Leptin concentration in rats (n = 6) submitted to 6h of advances every 5 days (advances) and control (T24). Blood samples were obtained at ZT12-13 (dark onset; OD), ZT18-19 (mid dark; MD), ZT0-1 (light onset; OL) and ZT6-7 (mid light; ML), where ZT12 is the beginning of the dark phase.
(TIF)

S2 Fig Histological examination of liver and epidydimal and subcutaneous WAT. H&E staining. Epi: epidydimal; sc: subcutaneous. (n = 7–8).
(TIF)

S3 Fig nNOS mRNA expression in the hypothalamus. nNOS mRNA levels in the hypothalamus. (*p<0.05, n = 7–8).
(TIF)

S1 Table Serum FGF21, NEFA, cholesterol and triacylglyceride (TAG) levels. (n = 7–8).
(PDF)
