# Supplementary figures and images for: Altered Circadian Rhythm and Metabolic Gene Profile in Rats Subjected to Advanced Light Phase Shifts
Source: PLoS One. 2015 Apr 2;10(4):e0122570. doi: 10.1371/journal.pone.0122570 (PMC4383616; doi:10.1371/journal.pone.0122570)

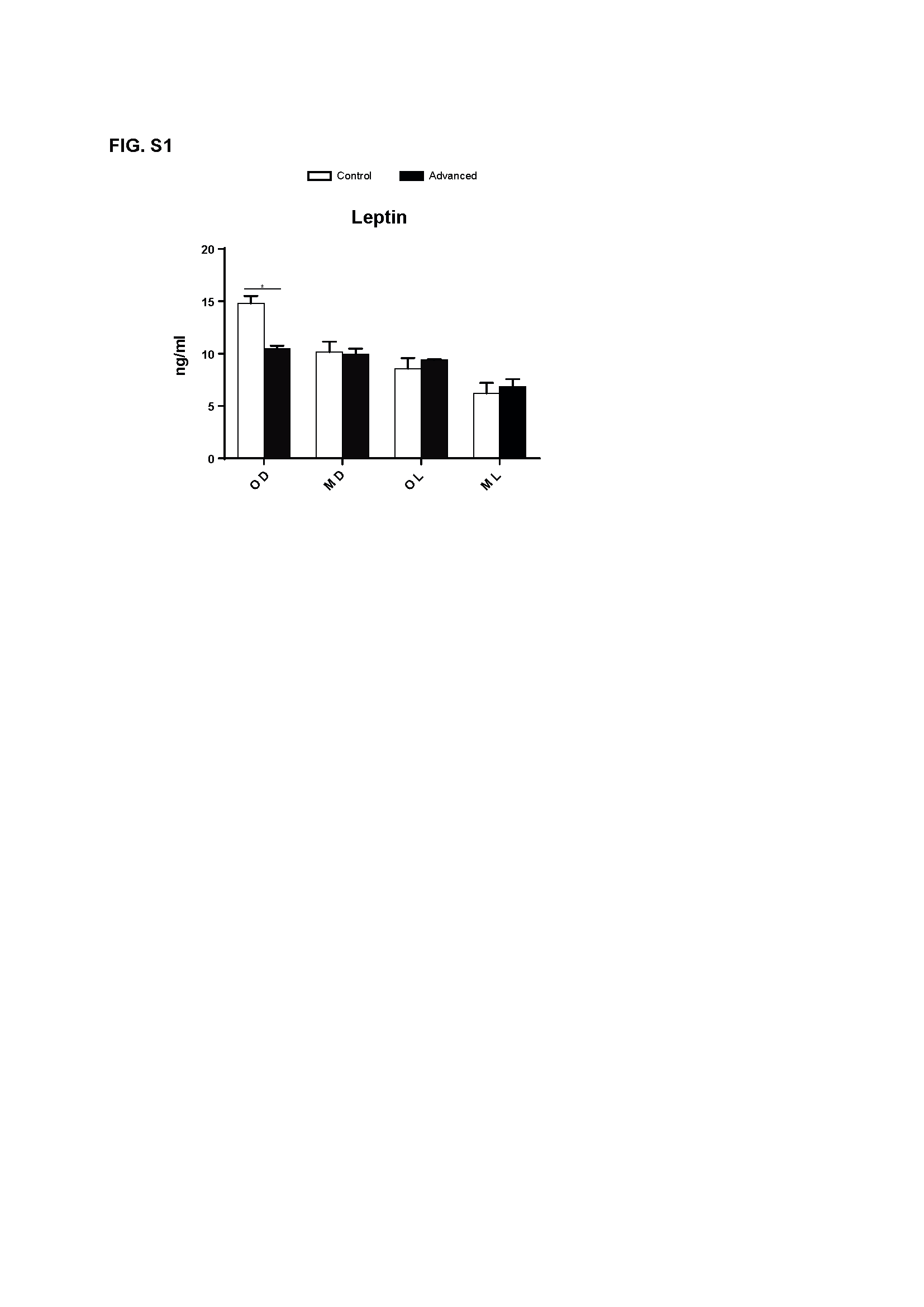

Supplement: S1 Fig — Leptin concentration in rats (n = 6) submitted to 6h of advances every 5 days (advances) and control (T24). Blood samples were obtained at ZT12-13 (dark onset; OD), ZT18-19 (mid dark; MD), ZT0-1 (light onset; OL) and ZT6-7 (mid light; ML), where ZT12 is the beginning of the dark phase. (TIF) [file pone.0122570.s001.tif]

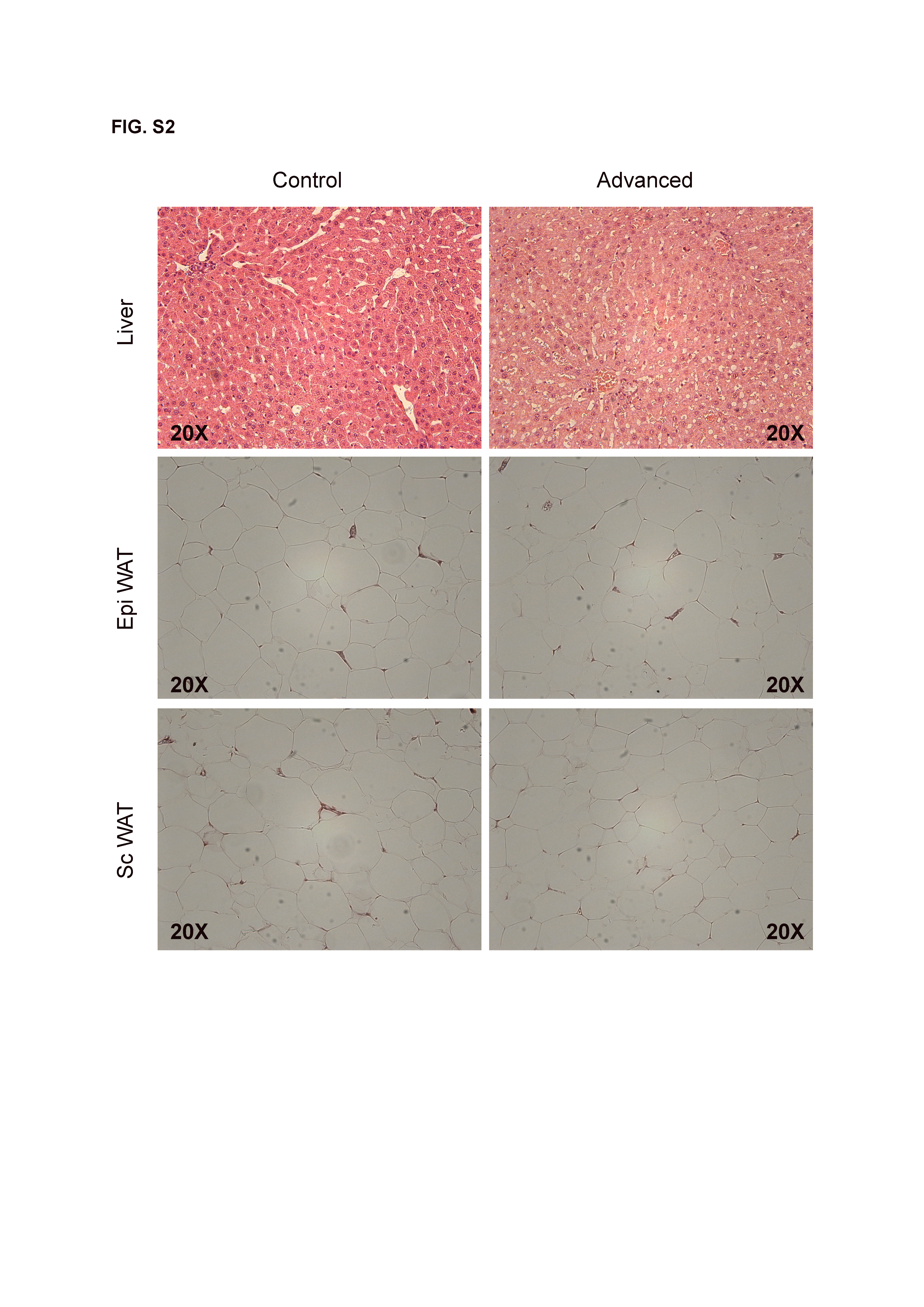

Supplement: S2 Fig — H&E staining. Epi: epidydimal; sc: subcutaneous. (n = 7–8). (TIF) [file pone.0122570.s002.tif]

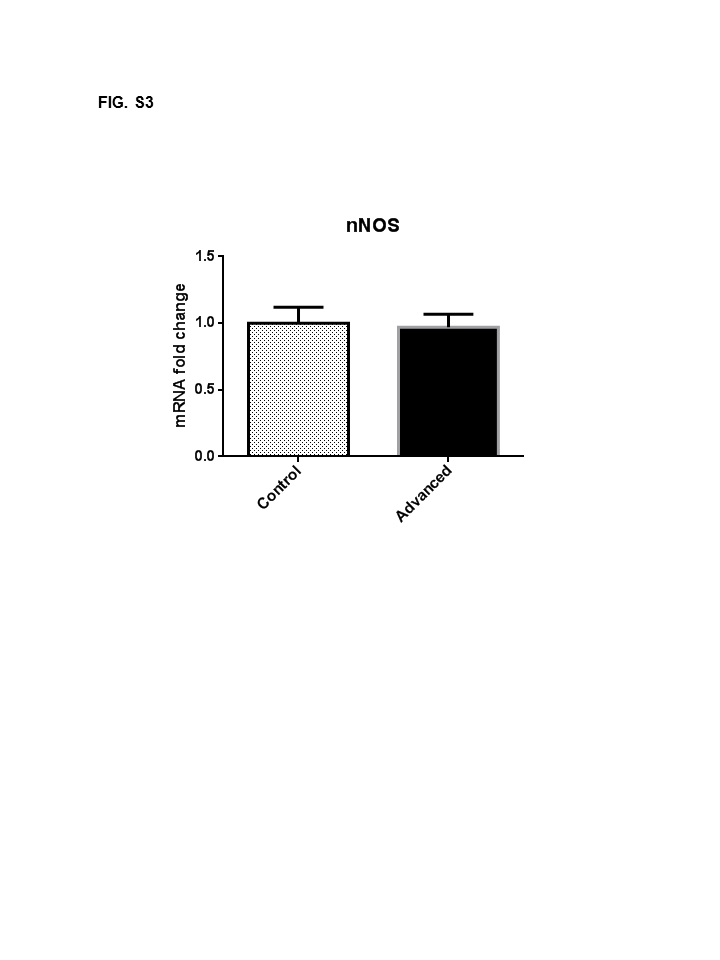

Supplement: S3 Fig — nNOS mRNA levels in the hypothalamus. (*p<0.05, n = 7–8). (TIF) [file pone.0122570.s003.tif]
