# Supplementary material for: Altered Circadian Rhythm and Metabolic Gene Profile in Rats Subjected to Advanced Light Phase Shifts
Source: PLoS One. 2015 Apr 2;10(4):e0122570. doi: 10.1371/journal.pone.0122570 (PMC4383616; doi:10.1371/journal.pone.0122570)
Supplement: S1 Table — (n = 7–8). (PDF) [file pone.0122570.s004.pdf]

S1 Table

	**Control**	**Advanced**
**FGF21 (U/ml)**	46.33 (± 4.7)	56.87 (± 12)
**NEFA (mM)**	1.09 (± 0.11)	1.17 (± 0.14)
**Cholesterol (mg/ml)**	86.16 (± 3)	81.58 (± 7.7)
**TAG (mg/ml)**	6 (± 1.3)	6.84 (± 1.05)
